# New Developments and Challenges in Liver Transplantation

**DOI:** 10.3390/jcm12175586

**Published:** 2023-08-27

**Authors:** Amjad Khalil, Alberto Quaglia, Pierre Gélat, Nader Saffari, Hassan Rashidi, Brian Davidson

**Affiliations:** 1 Liver Unit, Wellington Hospital, London NW8 9TA, UK; 2 Centre for Surgical Innovation, Organ Regeneration and Transplantation, University College London, London NW3 2PS, UK; 3 Clinical Service of HPB Surgery and Liver Transplantation, Royal Free Hospital, London NW3 2QG, UK; 4 Cancer Institute, University College London, London WC1E 6DD, UK; 5 Division of Surgery and Interventional Science, University College London, London NW3 2PS, UK; 6 Department of Mechanical Engineering, University College London, London WC1E 7JE, UK; 7 Institute of Child Health, University College London, London WC1N 1EH, UK; h.rashidi@ucl.ac.uk

**Keywords:** liver transplantation, history, limitations, organ preservation, ex vivo perfusion, transplant oncology, hepatocyte transplant, nonalcoholic fatty liver disease, regenerative medicine, cell therapy

## Abstract

Liver disease is increasing in incidence and is the third most common cause of premature death in the United Kingdom and fourth in the United States. Liver disease accounts for 2 million deaths globally each year. Three-quarters of patients with liver disease are diagnosed at a late stage, with liver transplantation as the only definitive treatment. Thomas E. Starzl performed the first human liver transplant 60 years ago. It has since become an established treatment for end-stage liver disease, both acute and chronic, including metabolic diseases and primary and, at present piloting, secondary liver cancer. Advances in surgical and anaesthetic techniques, refined indications and contra-indications to transplantation, improved donor selection, immunosuppression and prognostic scoring have allowed the outcomes of liver transplantation to improve year on year. However, there are many limitations to liver transplantation. This review describes the milestones that have occurred in the development of liver transplantation, the current limitations and the ongoing research aimed at overcoming these challenges.

## 1. Introduction

In 1952, Milan, Italy, Vittorio Staudacher performed the world’s first liver transplant in a large animal (canine) model. In humans, the first successful solid organ transplant was performed in 1954 with a kidney transplant between identical twins [1]. Subsequently, in March 1963, Thomas Starzl performed the first human liver transplant at the University of Colorado, on a 3-year-old boy with biliary atresia. The boy regrettably died during the operation as a result of haemorrhage [2]. Over the following 7 months, four further patients underwent unsuccessful liver transplants. Early results in other centres, including Boston, USA in 1963 and Paris, France in 1964, were also associated with short-term failure. Liver transplantation was still considered an experimental procedure with rather poor survival rates. In July 1967, Thomas Starzl performed the first successful liver transplant for primary liver cancer on a 1-year-old girl with hepatocellular carcinoma (HCC). She survived 13 months before dying as a result of metastases [2]. Other centres around the world began to adopt and pioneer liver transplant programmes modifying and improving the technical procedure and, as a result, gaining increasingly successful results. One of the first programmes established was in the UK. Roy Calne, a surgeon from Cambridge, and Roger Williams, a Hepatologist from Kings College, London, advocated for strong interhospital collaborations as a necessity for liver transplantation. They developed the U. K’s first liver transplant programme and performed the first human heterotopic (splenic fossa) and cava-preserving (piggyback) liver transplants [3]. These pioneers encouraged the development of liver transplantation throughout the world and piloted technical procedures many of which are still in use at present.

Deaths following liver transplants were subsequently mainly related to the effects of ischemic injury to the donor organs and reperfusion injury to the graft, leading to liver failure and/or sepsis. Ischaemia reperfusion (IR) injury is related to organ preservation damage between organ retrieval from the donor and implantation in the recipient. IR is characterised by hypoperfusion and hypoxia of the graft, leading to oxidative stress and a surge in pro-inflammatory cytokines, damage to cellular organelles and the formation of oxidative free radicals. This damage leads to apoptosis and necrosis of hepatocytes, cholangiocytes and sinusoidal endothelial cells in the graft, which clinically manifests as an increased risk of primary non-function, early graft dysfunction, biliary strictures and reperfusion syndrome manifesting in severe multi-organ failure in the recipient [4].

## 2. Key Developments

### 2.1. Brain Death Legislation

Liver transplant outcomes were greatly improved with legislation changes in 1968, through the establishment of the concept of ‘brain death’ or irreversible coma in the United States. This allowed the retrieval of organs from brain dead but ‘heart-beating’ donors in near normal physiological conditions, resulting in superior graft quality and function [5].

### 2.2. Drug Therapies for Immunosuppression

Drug developments have also transformed organ transplant. In 1979, the calcineurin inhibitor cyclosporine was introduced as part of the immunosuppressive regimen. It had a radical effect, reducing rejection, opportunistic infections and toxicity compared to the alternative immunosuppressants at the time, namely, azathioprine and steroids [6]. A dramatic improvement was observed in patients’ survival, and this success was further improved in 1987, with the introduction of tacrolimus (FK506), another calcineurin inhibitor, with low rejection rates, graft loss and significantly fewer side effects compared to cyclosporin [7]. Tacrolimus has continued to be the first-line immunosuppression for the majority of liver transplant patients. Mycophenolate mofetil (MMF), mammalian targets of rapamycin (mTOR) inhibitors (Sirolimus), and anti-CD25 (Basiliximab) were introduced as immunosuppressive adjuncts through the 1990s [8].

Immunosuppressants have a narrow therapeutic index with under treatment leading to rejection, graft damage and loss. Over treatment increases the risk of opportunistic infection, malignancy and drug-specific toxicity. The monitoring of both graft function and immunosuppression levels is required to reach the subtle balance between the two, when providing individualised therapy. Approaches to achieve the lowest therapeutic dose include the ‘bottom-up’ regimen, which either delays initiation and/or initiates immunosuppression at a lower dose; this is particularly beneficial in end-stage liver disease with renal dysfunction, though lacks supporting randomised trial data [9,10].

### 2.3. Organ Preservation Solutions

A successful attempt to tackle ischaemia reperfusion syndrome in donor organs and reduce cellular swelling originated in 1987, with the introduction of the University of Wisconsin (UW) solution by surgeon Folkert Belzer. This organ preservation fluid reliably extended the period that livers could be preserved ex vivo from less than 8 h to more than 15 h [11]. It allowed the transport of donor livers over long distances and reduced some of the urgency in the transplantation process into the recipient [12]. The UW solution also reduced the risk of primary non-function, early graft dysfunction and reperfusion syndromes [11]. Cold storage led to reduced metabolism reducing respiratory chain function, succinate accumulation and breakdown of ATP/ADP to purine metabolites. The UW solution became one of the most important factors in the evolution of organ transplant and remains the gold standard solution for the preservation of liver grafts at present [12].

### 2.4. Coagulation Control

Initial attempts at liver transplantation frequently led to massive blood loss and were a major cause of morbidity and mortality. Bleeding diathesis in liver transplantation is multifactorial and is associated with the need for the frequent use of both blood and blood products, including fresh frozen plasma, clotting factor concentrates and platelets. Causes of bleeding include the severity of pre-existing liver disease, portal hypertension, varices, abdominal adhesions and pre-operative blood count, but surprisingly not the degree of pre-operative coagulopathy or hypercoagulability. Additional factors related to blood loss include surgical technique, team experience, central venous pressure, duration of vascular clamping, duration of anhepatic phase, use of portocaval shunting, use of both anticoagulants/prothombotic agents and use of thromboelastography through point-of-care testing (POC) [13]. As liver transplantation programmes have increased, so has the demand on blood services. Considerable blood transfusion requirements negatively impact clinical outcomes, including the length of hospital stay and patient and graft survival. Methods to reduce transfusion requirement have included reducing central venous pressure, using blood products (such as fresh frozen plasma, cryoprecipitate and platelets), haemodilution and use of intra-operative blood salvage autotransfusion. As a result of these improvements, it is not uncommon for liver transplants to be performed without the need for blood transfusion [14].

### 2.5. Donor after Brain Death (DBD) and Donor after Circulatory Death (DCD)

Liver transplantation prior to the development of the Harvard criteria for brain death in 1968 relied exclusively on non-heart-beating donors (NHBDs) prior to organ retrieval and, as such, all were DCD (donated after cardiac death) liver transplants [15]. From 1968 to the 1990s, the majority of livers implanted were from DBD (donated after brain death) donors. In 1995, an expert work group working on the NHBD criteria met in Maastricht and standardised the ‘Maastricht classification’ of organ donation after circulatory arrest with five categories, defined as category I (arrived already deceased to hospital), category II (unsuccessful resuscitation), category V (unexpected arrest, whilst in intensive care) as uncontrolled circulatory arrest, category III (life-sustaining treatment withdrawn due to futility of further care) and category IV (cardiac death after brain death). Categories III and IV are considered controlled circulatory arrest in which the retrieved organs are less likely to suffer from complications related to ischaemic injury [15]. From 1995 to 2007, DCD liver transplants increased dramatically, with initial reports describing favourable outcomes with DCD livers from NHBDs with controlled circulatory arrest. As donor demand grew, the utilisation of marginal/higher risk grafts grew (extended criteria donors). Organ availability improved but was associated with increasing complications [16]. DCD liver transplantation saw a decline between 2007 and 2012 with higher rates of complications emerging [15]. Since 2012, with improvements in donor graft selection and selection of recipients more tolerant to the effects of initial graft dysfunction, DCD liver transplants have continued to increase with satisfactory outcomes, representing 23% of livers transplanted in the UK in 2022 [17].

### 2.6. Living Donor Liver Transplantation (LDLT)

Despite the benefits to organ transplant outcomes observed with the use of the brain death criteria in the West, this did not become culturally accepted in many Middle Eastern and Asian countries, such as Japan and Korea, despite establishing laws in support of brain death. As a result, a lack of donors remained a major limitation to organ transplantation in Asia [18].

Transplant in Asia increased considerably following the introduction of a technique founded by Henri Bismuth, from France, in which a whole adult liver was reduced to just the left lobe and implanted in a child [19]. Rudolf Pichlmayr, from Hannover, Germany in 1988, continued upon this success with the development of the split-liver transplant, dividing a single allograft liver for implantation into two separate recipients [20]. This laid the foundation for partial hepatectomy, or removing part of the liver from a living donor and transplanting this portion into a recipient with liver disease. Russel Strong, from Brisbane, Australia, pioneered the first LDLT in 1990 [21].

LDLT became a valuable resource to address the lack of donor grafts, predominantly utilising full right, left or left lateral grafts, but also including a diverse array of liver graft types, such as right anterior, right posterior and even extended lobe grafts, (trisection +/− caudate lobe) [22,23].

Living donors are generally healthy volunteers who receive no medical benefit from the transplantation, so ensuring low morbidity and mortality is of paramount concern [24]. Pre-operative assessments include, but are not limited to: donor cross-sectional imaging for the visualisation of aberrant vascular and biliary anatomy, liver fat quantification with MR (fat fraction) or biopsy (steatosis grade/%), and liver volumetry, including estimated remnant liver volume and estimated graft-to-recipient weight ratio (GRWR) to reduce the risk of small-for-size problems in both the donor and recipient [25].

Donor mortality is reported at 0.2%, whilst morbidity is in the range of 20–40%. To further minimise the extent of surgical trauma and shorten donor recovery, the original large open Mercedes incision evolved to a reverse L incision and subsequently a small upper-midline incision [26].

In 2002, in France, Daniel Cherqui reported the first laparoscopic living donor left lateral sectionectomy from a parent to their 1-year-old infant recipient with biliary atresia [27]. In 2006, in Chicago, USA, Koffron performed a laparoscopic-assisted right lobe donor hepatectomy; in 2010, in South Korea, Han performed the first pure laparoscopic donor right hepatectomy; and in 2013, in Ghent, Troisi performed the first pure laparoscopic left hepatectomy. During this period of minimally invasive approaches, Giulianotti performed the first right-lobe robotic living donor hepatectomy in Chicago, in 2012 [28]. Hybrid approaches, utilising both laparoscopic donor hepatectomy and robotic implantation, have been performed since 2022. Operative and ischaemic times have doubled in some reported cases of minimally invasive surgery, which can have deleterious effects on the graft and patient and, as such, should only be performed in selected cases [25].

#### Auxiliary Liver Transplantation

Similar transplant feats included the auxiliary partial liver transplant technique, which built upon heterotopic implantation or more commonly liver reduction surgery and the implantation of a partial liver graft in an orthotopic position. This auxiliary technique for acute liver failure relies upon the graft to support liver function until the native liver recovers. With the recovery of the native liver, immunosuppression can be discontinued, with the subsequent surgical removal or atrophy of the auxiliary graft. A two-staged auxiliary technique was also developed for end-stage liver disease, i.e., native liver reduction surgery with the implantation of a smaller left lobe graft, performed in stage one. After the graft, liver hypertrophy and GRWR increase, a second procedure is performed with the complete removal of the diseased native liver, reducing the risk of small-for-size problems and hepatic artery thrombosis. The left lobe is the preferred donation option as it is associated with fewer donor complications than right lobe hepatectomy [29].

### 2.7. Hepatocyte Transplant

Human hepatocyte transplantation was introduced in Japan in the early 1990s as an alternative to whole-organ transplant, with hepatocytes being the major functional component of the liver. Hepatocyte transplant could be performed repeatedly, and multiple patients could be treated with cells from a single donor organ. Hepatocytes could be cryopreserved, allowing them to be available when required. The transplanted hepatocytes could support the function of the native liver, allowing it to remain in situ and provide background functioning of the native liver in the event of donor hepatocyte graft failure. The preservation of the native liver would also allow for a target for future therapies, especially in the treatment of metabolic liver disorders [30].

The initial experience of a human hepatocyte transplant consisted in the autologous transplantation of cells harvested from the left lateral segments of the liver in a ten-patient cohort, all of whom had liver cirrhosis, with the aim of providing metabolic support [30]. Cells were infused via alternate routes, including direct splenic puncture, portal vein and splenic artery injections; four patients also underwent ligation of the hepatic artery. Due to the inconsistent approaches and heterogeneity, the intended benefits were difficult to determine [30]. In addition, hepatocyte transplant in portal hypertension and portal–systemic shunting increases the risk of portal vein thrombosis and pulmonary embolism via the translocation of hepatocytes into the pulmonary circulation [31]. Observable benefits are yet to be established for hepatocyte transplantation in the presence of cirrhosis.

In contrast, for the management of metabolic liver disorders in paediatric patients, hepatocyte transplant has a proven safety record, with a moderate therapeutic profile. The replacement of hepatocyte enzyme activity is what provides benefits in metabolic disorders. A modest 100 million hepatocytes/kg body weight or the cell replacement of 5–10% of the total liver mass can provide temporary therapeutic effects lasting several months. In such cases, hepatocytes are infused via the portal vein [32]. The intravascular infusion of hepatocytes is inefficient, with clearance by Kupffer cells and the majority of hepatocytes remaining wedged in portal spaces [33].

Hepatocyte transplant has also become a ‘bridging’ therapy for acute liver failure; this approach requires a larger number of hepatocytes to achieve a critical mass and aims to inject 10–15% of functioning liver cells. The portal vein is generally avoided, and alternative sites, such as the peritoneal cavity, are targeted instead [33]. The efficacy of hepatocyte transplant is difficult to evaluate in the absence of randomised trials. Further difficulties with assessing efficacy in acute liver failure include the fact that 20% of patients with acute liver failure recover without transplantation. Despite designated centres and guidelines for hepatocyte transplant, this technique remains confined to clinical research rather than being the standard practice for the treatment of acute liver failure.

### 2.8. Machine Perfusion

The idea of maintaining the viability of organs outside the body with artificial circulation was suggested as early as 1812 by the physiologist Le Gallois [34]. Through the 1800s, physiologists were able to demonstrate the production of urea by an ex vivo liver connected to a circulation apparatus. Assessing the viability of organs was limited. In 1935, Alexis Carrel and Charles Lindbergh invented a perfusion pump (the Carrel–Lindbergh perfusion pump) as an apparatus to maintain organ viability. It mimicked blood circulation utilising a pump, rotary valve, filter and glass tubing, creating a pulsatile pressure to perfuse organs aseptically [34].

#### 2.8.1. Hypothermic Machine Perfusion (HMP)

Folkert Belzer, the pioneer of the University of Wisconsin solution, developed and successfully used machine perfusion in 1968, starting with kidney transplantation on a canine model [35]. The initial design consisted of a membrane oxygenator with a pulsatile pump, organ chamber and arterial and venous reservoirs in hypothermic temperatures. Hypothermia slowed down the metabolic activity and the degradation process, and had the advantages of preserving microcirculation and thus the peribiliary vascular plexus, delivering nutrients and oxygen continuously to meet metabolic demands, whilst also removing the build-up of metabolic waste products [36]. Due to the logistical challenges involved in utilising hypothermic machine perfusion (HMP), its use was abandoned in 1970/80s in favour of static cold storage (SCS). However, it has since seen a re-emergence due to the limitations of SCS for marginal donor grafts and the development of improved perfusion systems.

#### 2.8.2. Normothermic Machine Perfusion (NMP)

NMP was introduced to mimic in vivo conditions. NMP provided the option to assess aspects of graft function, such as bile output, lactate clearance, glucose metabolism and transaminase levels prior to transplanting. Several clinical trials comparing SCS and machine perfusion demonstrated major benefits in improved organ utilisation, organ viability testing, reduced organ injury and longer periods of preservation [37].

### 2.9. Liver Transplantation for Viral Hepatitis

#### 2.9.1. Hepatitis C (HCV)

In 1978, a highly infectious and transmissible transfusion-associated hepatitis was recognised in the clinical setting. At the time, the infection was labelled as non-A, non-B hepatitis (NANBH). It was only in 1989 that the hepatitis C virus (HCV) was first identified as the causative agent and, not long after its identification, the first diagnostic HCV antibody test was developed [38]. HCV caused a massive global burden leading to chronic liver infection, cirrhosis and HCC and accounted for a large proportion of the liver transplants performed (30–45% in US and Europe) with initial poor patient and graft survival compared with other liver transplant indications. Prior to the introduction of effective anti-virals, reinfection was almost universal with resultant damage to donor organs as soon as 3 months post-transplantation and progression to cirrhosis at 5 years as high as 20–30% [1]. Three years prior to the identification of HCV, interferon-based therapy (IFN) had been used to treat NANBH. In 1991, a nucleoside analogue, ribavirin (RBV), was initiated as HCV treatment and, by 1994, IFN and RBV became dual therapy in the treatment of HCV. In 2003, the first direct-acting antiviral agent (DAA) was tested in humans, but it was not until 2011 that DAAs were approved. By 2015, they were used to treat HCV prior to transplantation, improving liver transplant outcomes in this cohort. The introduction of DAA therapy was ground-breaking, with 98% of patients infected with HCV having a sustained virological response (SVR) after a 12-week course of treatment [38]. The success of HCV treatments has not only reduced the need for liver transplantation but has also led to the acceptance of donor organs from HCV-infected patients for use in transplantation.

#### 2.9.2. Hepatitis B (HBV)

Despite the remarkable advancements in the treatment of HCV, chronic infection with hepatitis B (HBV) is contracted in 400 million people globally [39]. HBV is an enveloped, partially double-stranded, hepatotropic, circular DNA virus, with chronic infection causing continued hepatocyte damage leading to fibrosis, with the development of cirrhosis and primary liver cancer in up to 25% of infected patients [40]. HBV is implicated in the death of over 800,000 people annually [41]. Despite its ongoing major public health impact, the incidence of HBV is on the decline in many parts of the world, mainly as a result of the rollout of HBV vaccination programmes in the mid 1990s [39]. Treatment with reverse transcriptase inhibitors and IFN reduce the circulating HBV viral load, thereby reducing the risk of transmission and, as such, has also facilitated the decreased incidence of HBV [41].

The primary indication for liver transplantation in chronically infected HBV patients is HCC on a background of cirrhosis. Only a minority have decompensated cirrhosis as a result of effective antiviral therapy [42]. Post-liver transplant therapy commonly consists of a combination of hepatitis B immunoglobulin and reverse transcriptase inhibitors with the recurrence of HBV in less than 10% of transplanted patients [43].

### 2.10. Transplant for Cancer

Indications for liver transplant have evolved over the years. In 1996, the Milan criteria were established for selecting patients with HCC for transplant. The criteria improved cancer-free survival from 30% (with liver resection) to 75% (with liver transplant) at the cost of major surgery and lifelong immunosuppression [44]. Whilst there were clearly demonstrable benefits to the Milan morphological criteria, these only indicated liver transplant for 30% of those afflicted with HCC. Multiple additional criteria have since emerged, broadening the scope of liver transplantation for HCC, particularly in high-volume LDLT centres incorporating biological and serum parameters alongside morphological characteristics [45].

Liver transplantation has long been a treatment for primary liver cancers, such as hepatoblastoma, epithelioid hemangioendothelioma and HCC (as discussed above) [46]. Liver transplant for cholangiocarcinoma and secondary liver cancers was initially associated with high recurrence rates, with most immunosuppressive agents considered to accelerate tumour spread [47,48].

Liver transplantation became a recognised treatment for neuroendocrine (NET) hepatic metastases after clinical trials demonstrated long-term graft and patient survival comparable with patients transplanted for HCC [46]. Clinical studies have also shown good outcomes and survival rates in selected patients combining multimodal therapies of neoadjuvant chemotherapy, radiotherapy and liver transplantation as a treatment indication for unresectable hilar or perihilar cholangiocarcinoma and intrahepatic cholangiocarcinoma on a background of primary sclerosing cholangitis (PSC) [46].

The Norwegian SECA trials that started in 2006 demonstrated that liver transplantation provides superior survival rates in non-resectable colorectal liver metastases to standard cancer therapies and has survival rates comparable with other indications for liver transplant at 5 years [49]. Pilot clinical evaluations utilising liver transplant for cancer are currently being held throughout Europe to demonstrate whether these early outcomes can be reproduced.

The increasing prevalence of malignant liver disease and utilisation of liver grafts for its treatment will place an increased burden on transplant services, with liver transplant demand exceeding the supply of donor organs. This is balanced in part by the reduction in the need for transplantation for HCV due to the success of antiviral therapies. Allocating organs for cancer therapy will require balancing oncological disease recurrence under immunosuppressive therapy with liver allocation shortages for patients with non-malignant liver disease, which poses a challenging dilemma.

Within 30 years of the first liver transplant mortality, the overall liver transplant survival rates have improved to >90% and 85% at 1- and 5-years post-transplant, respectively [50,51].

### 2.11. Improving the Diagnosis of Organ Rejection: The Banff Classification

Despite the wide success of liver transplantation and improved immunosuppression, acute liver rejection is present in up to 25% of patients early following transplant, contributing to significant morbidity [52]. Pathological changes associated with acute and chronic rejection have been reported in the literature from the late 1960s. Despite the correct histological interpretation of rejection having important implications on patient management and outcomes, there remained no gold standard in diagnosing rejection [53]. A number of grading systems utilising semi-quantitative assessments had been in use, including Birmingham, European, Minnesota, Pittsburgh and The Royal Free Hospital grading systems [54]. In 1991, a group including a renal pathologist, nephrologist and transplant surgeons met in Banff, Canada, and established the Banff classification of allograft pathology, which was an international standardised classification for the definition and grading of rejection from solid organ transplant biopsies. The Banff system refined the grading into two parts, global assessment (GA) and rejection activity index (RAI), with the total score converted to rejection grades, from nil to severe [54]. In 1995, in Banff, Canada, a group of liver transplant, including a hepatologist, surgeons and a pathologist, extrapolated this concept and agreed on a histopathological criteria for grading of acute liver rejection [55]. Subsequent Banff meeting reports updated the criteria for the histological assessment of chronic rejection [56], late allograft dysfunction [57], operational tolerance [58], antibody-mediated rejection [59,60] and donor liver steatosis [61].

### 2.12. Organ Allocation Prioritisation

**Figure 1:**
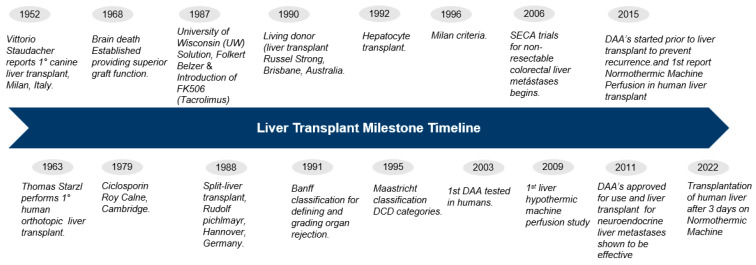
Liver transplant milestones. Abbreviations: DCD—Cardiac death; DAA—Direct-acting antiviral agent.

The Model For End-Stage Liver Disease (MELD) was originally developed as a reliable predictor of short-term survival in patients with end-stage liver disease undergoing transjugular intrahepatic portosystemic shunt (TIPS) [62]. By 2002, MELD was utilised for liver priority allocation. In 2016, the US changed the scoring model to MELD-sodium (MELD-Na) [62], and in 2023, the MELD 3.0 became the predictor of choice for liver priority allocation, taking account of changes with the epidemiology of patients with liver disease, prognosis and comorbidities. The previous MELD scoring underestimated short-term survival in females, with serum creatinine overestimating renal function, making it less likely for females to receive a transplant compared to male counterparts with identical scores. Smaller abdominal cavities generally found in females also restrict the suitability of larger donor grafts [63] (Figure 1).

## 3. Current Limitations to Liver Transplant

### 3.1. Limited Availability of Organ Transplantation

Although liver transplantation is seen as a major success story, there remain major limitations associated with this procedure. Patients in need of a liver transplant require referral to a transplant centre, evaluation of suitability for receipt of a liver transplant and registration on a transplant waiting list. They must also be fit enough to survive until a suitable donor graft is allocated.

According to the Global Observatory on Donation and Transplantation (GODT), transplantation only covers 10% of the global need. This mainly results from the limited availability of transplant centres and expertise. A study in the United States found only 21% of patients fulfilling referral criteria for a liver transplant were transplanted [64].

### 3.2. Transplant Organ Shortage

There is also a shortage of organ donors worldwide, with 15% of patients dying whilst on the liver transplant waiting list [65]. Available donor grafts have become a major limiting factor with demand exceeding supply. Priority allocation protocols and registers have been designed and employed, such as the United States, MELD and the United Kingdom Model For End-Stage Liver Disease (UKELD), both of which are used as a method of grading the disease severity of recipients and prioritising those most in need [1]. In the UK, donor grafts are considered a national rather than a local resource and, as such, are controlled through a central recipient registration and donor allocation service.

### 3.3. Use of High-Risk Donor Organs

In an effort to increase the donor pool and meet the growing demand over the last 20 years, extended criteria donors have been utilised, retrieving and implanting grafts from marginal/higher risk donors, such as older donors with comorbidities, and donors after circulatory death (DCD). The high-risk donors increase donor organ availability but have a greater susceptibility to ischaemic reperfusion injuries, resulting in increased morbidity and mortality [16].

This has necessitated the adoption of innovative technologies and strategies to protect these higher-risk grafts from the deleterious effects of traditional preservation methods and ischaemia reperfusion injury (IRI) [66]. To improve the outcomes of these expanded donor grafts, much investment and research has been devoted to applications such as normothermic and hypothermic machine perfusion. Although initial studies suggest that there are benefits associated to these technologies, there is a major impact of cost and technical support required, whilst the evidence of patient-centred benefit remains unclear. Further trials are required to conclusively evaluate this. Normothermic machine perfusion is yet to demonstrate patient benefits, including reduced mortality, reduced hospital stays, prolonged graft and/or patient survival [67]. The perfused liver utilisation (PLUS) study is currently evaluating if normothermic machine perfusion (NMP) improves organ utilisation without compromising outcomes in livers with extended criteria. This topic is discussed more extensively elsewhere in this Special Issue [68].

Hepatocyte transplant has demonstrated short-term success and has an established safety profile for selected patients with acute liver failure and some liver-based metabolic disorders. Its use is limited by the shortage of healthy cells, combined with apoptosis and persistent decline in function with prolonged culturing, low engraftment and a reduced proliferative capacity, which has prevented its use in most situations.

### 3.4. Cost of Liver Transplantation

Due to the large financial and logistical costs, liver transplantation barriers remain, with disparities disproportionately effecting low-income countries. In 2017, one liver transplant admission was estimated to cost USD 463,200 and CAD 102,597 in the USA and in Canada, respectively, both excluding physician costs [69]. The associated high cost, highly skilled teams, infrastructure and peri-operative care prevent its application in many low-income countries.

### 3.5. Obesity and Non-Alcoholic Steatohepatitis (NASH)

The global obesity epidemic along with associated metabolic syndrome have led to non-alcoholic steatohepatitis (NASH) as the most common indication for liver transplantation in females and will likely become the same for males [1]. The epidemic of NASH also has a knock-on effect by decreasing the donor pool of acceptable grafts and increasing the strain on transplant services with high-risk donors and recipients. Metabolic syndrome manifest itself in the liver with non-alcoholic fatty liver disease (NAFLD) and affects 25% of the population worldwide; a total of 25% of those with NAFLD will suffer from the progressive inflammatory NASH subtype [70,71]. More than half of liver transplant patients are classified as obese or morbidly obese, and this is set to rise [72]. Obesity in the transplant setting comes with multiples challenges. The procedure is technically more challenging with increased surgical times and surgical complications [70]. Obesity is independently associated with an increased risk of cancer, cerebrovascular disease, diabetes, dyslipidaemia, renal impairment, pulmonary impairment and PV thrombosis [71,72]. Understandably, cardiovascular events are the most common cause of death in this cohort [71].

The mainstay of non-liver transplant treatment is at present lifestyle changes with weight loss from dietary modification and exercise. There are multiple phase-3 drug trials for the treatment of NASH and for weight loss, but as yet there is no evidence demonstrating benefits in end-stage liver disease patients [71]. While bariatric surgery for weight loss is a feasible treatment adjunct in the liver transplant algorithm, with more reliable weight loss and fewer metabolic complications compared to liver transplant alone [71,72], the ideal timing (simultaneous v sequential) remains undecided [71,72]. Procedures in order of invasiveness include gastric balloons (non-operative), gastric banding, sleeve gastrectomy and gastric/ small bowel bypass [72]. Gastric banding is a minimally invasive, short procedure, which is relatively safe and does not affect endoscopic access to the biliary tree; however, it is associated with poor weight loss efficacy, foreign body infection and risk of band migration [70,72]. Sleeve gastrectomy (SG) as an adjunct to liver transplantation has the strongest of the limited evidence amongst bariatric operations, with a good balance between safety and efficacy [72]. SG does not cause malabsorption and does not affect pharmacokinetics of immunosuppressants or affect endoscopic access to the biliary tree, but it does have an increased risk of staple-line bleeding and leak. Roux-en-Y gastric/small bowel bypass has the greatest efficacy for weight loss, although it is the most invasive and has the longest duration of any of the other procedures. It can affect endoscopic access to the biliary tree and the pharmacokinetics of immunosuppressants and is associated with the highest risk of malabsorption and sarcopenia of all the bariatric procedures [70,71,72].

#### 3.5.1. Bariatric Surgery Pre-Liver Transplant

Potential benefits of pre-liver transplant bariatric surgery include weight loss with an intention to be listed in LT centres with BMI limits, improvement in liver function and reduction in obesity-related complications [72].

There are higher risks of bariatric-surgery-related complications, including hospital stay, morbidity and mortality rates, in decompensated and compensated cirrhosis compared to non-cirrhotic patients. Other risks include rapid malabsorption and sarcopenia, which are associated with worse liver transplant outcomes than pre-existing obesity [72].

#### 3.5.2. Bariatric Surgery during Liver Transplant

Simultaneous bariatric surgery and liver transplant allows for a single operation and recovery phase. Patients who underwent simultaneous bariatric surgery with liver transplant had significantly better weight loss, graft steatosis and blood sugar control compared to patients undergoing liver transplant alone. Patients during post-bariatric surgery may also benefit from a better control of immunosuppressant side effects [72].

A simultaneous approach has an increased operative time in a relatively unstable patient, increased risk of poor healing, infection and leak, due to immunosuppression. Immunosuppression dosing with rapid weight loss can be troublesome, with an increased risk of osteopenia and sarcopenia [72].

#### 3.5.3. Bariatric Surgery Post-Liver Transplant

Bariatric surgery post-liver transplant has potential benefits, especially in a stable non-cirrhotic patient without portal hypertension. It can result in significant weight loss, preservation or improvement of the transplanted graft function and improvement or resolution of obesity-associated conditions.

This approach has an increased risk of post-liver transplant adhesions, infection and graft rejection (particularly if performed during first year), poor wound healing and leak due to immunosuppression (steroid use is an independent risk factor for increased morbidity and mortality post-bariatric surgery) [72].

Further, information regarding strategies aimed at tackling the obesity epidemic are outside the scope of this review. The current mainstay for weight loss in the liver transplant setting is lifestyle changes. Larger randomised trials are needed to determine tolerability and timing of bariatric surgery in the liver transplant setting [72,73,74].

Additional liver transplant limitations also include the low yield of donated organs becoming definite transplants, disease recurrence, the need for lifelong immunosuppression accompanied with a risk of de novo cancer, progressive graft fibrosis (associated with a loss of both graft and life) and cardiovascular, renal and bone diseases.

## 4. Developing Strategies in Liver Transplant

### 4.1. Reducing Transplant Organ Requirements: Artificial and Bioartificial Liver Support Devices

During the 1990s, extracorporeal liver assistance devices were developed as a bridging therapy to prevent patients with acute liver failure from dying prior to transplantation and for supporting liver function until native liver recovery. These systems were developed to assist the function of the liver in both acute and chronic liver failure [75].

Artificial non-biological systems were based on the general concept of albumin dialysis and the removal of albumin-bound toxins from blood and/or plasma that accumulate during liver failure. These toxins are thought to be implicated in the sequelae of multiorgan dysfunction, including hepatorenal failure, hepatic encephalopathy, haemodynamic failure and immunodeficiency [76].

Protein-bound bilirubin and water-based molecules and toxins, such as ammonia, urea, creatinine and some cytokines, were also cleared. The most common systems used were the Molecular Adsorbent Recirculating System™ (MARS™), The Single-Pass Albumin Dialysis System (SPAD) and The Fractionated Plasma Separation and Adsorption System (Prometheus™) [75].

Bioartificial devices have a complicated design. In addition to artificial membranes for the clearance of toxins, they also contain bioreactors with a minimum of 10^10^ cryopreserved functional hepatocytes to assist liver regulative and synthetic functions. Hepatocytes are sourced from various methods, including traditional liver donor allografts, induced pluripotent stem cells and the most commonly used porcine-derived hepatocytes. Two well-known systems that showed promise were the Extracorporeal Liver Assist System (ELAD^®^) by Vital therapies and HepatAssist^®^ by Alliqua, but both systems failed to demonstrate significant improvements in the survival rate in liver failure [76].

### 4.2. Organ Assessment and Recovery Centres (ARCs)

Developments in transplantation have focused on the preservation of donor organs in the state they were explanted and mitigating their degradation process. Current strategies alongside bioengineering approaches are targeting ex vivo therapeutics of donor organs to attempt to create grafts that are superior to the state in which they were initially discovered. Lung Bioengineering 1 (LB1) is one such pioneering commercial company that repairs lungs for clinical transplantation. LB1 and its partner LB2 alone have the capacity to potentially cover the entire continents’ lung perfusion needs [77]. The idea for these organ recovery centres would be to exploit the opportunity to apply diagnostics and therapeutics to marginal grafts during ex vivo organ perfusion, including livers modified by stem cell therapies, gene modification, manipulation of immune responses and to use pharmacological and biological precision therapies [78]. In the UK, research and investment into ARCs is a major development and is described as an aspiration of the national (NHSBT) transplant strategy with donor livers as a key target [79].

### 4.3. Liver Scaffolds

Decellularised livers from both discarded human and xenograft livers can be used as a scaffold to preserve the microstructures and their associated microenvironment whilst eliminating cellular components and preserving the extracellular matrix and its bioactive properties. These structural components are required for engraftment, proliferation, differentiation, neovascularisation and the subsequent development of newly generated livers. Various methods to decellularise the liver exist, including physical, chemical and enzymatic. Unfortunately, none of these methods completely decellularise the scaffolds without damaging the extracellular matrix (ECM). The re-vascularisation of the scaffolds is a major challenge for biocompatible scaffolds and, whilst progress has been made in recreating the macrovasculature of the liver, the decellularisation of endothelial lining and the creation of the microvasculature in the hepatic sinusoids has been a major challenge [80]. The liver is made up of over 100 billion functioning hepatocytes, all within 50 microns of the microvasculature; liver scaffolds are thus limited by poor perfusion and the resultant ischaemic injury [81]. Unfavourable immune response and thrombogenicity are also aspects requiring improvement [80].

### 4.4. Cell Therapy for Liver Failure

Primary hepatocytes, embryonic stem cells (ESCs), foetal cells and induced pluripotent stem cells (iPSCs), along with supporting mesenchymal stem cells (MSCs), can be utilised for recellularisation purposes with or without liver scaffolds [79]. Pluripotent stem cells can self-renew and differentiate into all cell types in the body, promising an unlimited supply for human tissue regenerative applications [82]. In 2006, Shinya Yamanaka successfully reprogrammed adult somatic cells into pluripotent stem cells employing four reprogramming factors; Yamanaka’s technique has been studied and utilised since then. Hepatocyte-like cells (HLCs) are iPSCs generated by reprogramming somatic cells, and HLCs can be generated in two-dimensional structures that display both adult and foetal features or a more phenotypically stable three-dimensional, spheroid structure, such as hepatospheres [82]. Studies of transplanted iPSCs have demonstrated therapeutic efficacy in murine models with chronic liver disease [81], post-partial hepatectomy and for the treatment of metabolic liver disease [82]. Information regarding long-term safety and clinical efficacy are still very limited. Limitations regarding foetal phenotyping and viability have had some success in pre-clinical studies [82]. Concerns remain regarding low reprogramming efficiency and tumorgenicity. Cellular therapies may become superseded by in vivo gene therapy and nanomedicine, which have demonstrated particular benefit for the treatment of multiple monogenic liver diseases.

### 4.5. Gene Therapy for Metabolic Liver Disease

Since the 1990s, gene therapy (GT) has been investigated for the treatment of metabolic liver diseases, which are often monogenic and inherited and require the replacement of a missing enzyme activity to restore defective pathways. GT can be delivered ex vivo or in vivo. Ex vivo delivery requires the extraction of host cells, culturing, amplification, modification with a vector and reinfusion back into the host. In vivo delivery is delivered locally or systemically via non-viral or viral vectors, such as recombinant adeno-associated virus (rAAV) [83]. In vivo delivery is the current treatment of choice for hepatic disorders due to difficulties with hepatocyte manipulation ex vivo [84]. Hundreds of rare inborn monogenic liver disorders exist, often as a result of a single protein deficiency. Hepatocytes can be specifically targeted using vectors by three main methods: the genetic repair of a mutation to restore the correct version of a protein, genetic addition for the expression of a missing protein and gene silencing for the elimination of or reduction in accumulated toxic metabolites [84]. Its use in clinical trials has been marred by several major setbacks, including the death of a patient from systemic inflammatory response syndrome and the development of leukaemia in patients treated with GT [83]. GT has since shown promise in clinical trials with FDA approval for conditions such as haemophilia B [81]. Despite the promising trial data and potential benefits, challenges remain with transient gene expression, high cost, risk of tumorgenicity and severe adverse reactions, such as hepatotoxicity, neurotoxicity and severe immune reaction to higher doses of viral vectors [78]. Strategies to block the inhibitory action of humoral and cell-mediated immunity towards these gene vectors are currently being investigated [84]. Further information is outside the scope of this review, and further discussion can be found in the following recent review [85].

### 4.6. Immuno-T-Cell Therapy

Another Achilles heel of organ transplantation is immune tolerance and the need for lifelong immunosuppression. Current immunosuppression drugs are non-specific and act as either general immunosuppressants or by blocking inflammatory cascades. As such, these treatments are not a cure and are required to be administered lifelong. Regulatory T cells (Tregs) have long been established as a key to regulating immune response and thereby prevent organ rejection. Several immunotherapy approaches are utilised to increase Tregs numbers, such as in vivo IL-2-based expansion and cell-based adoptive transfer, including cell-engineered, polyclonal and antigen-specific approaches. Multiple studies have used the adoption of Tregs for tolerance induction in transplantation but have yet to show clinical benefits [86]. Table 1 summarises emerging strategies in liver transplantation and their prospective advantages and current limitations.

## 5. Conclusions

In only 60 years, liver transplantation has become an established and effective treatment for both acute and chronic liver failure. However, the cost and complexity of management has resulted in treatment being available to only a minority of those who will benefit from it. The increased demand for transplant must be matched with an increased donor organ availability, including strategies for preventing or reducing organ preservation and reperfusion injury, of which ex vivo hypothermic or normothermic machine perfusion are currently being most intensively assessed. New transplant indications include transplant for bile duct cancer (cholangiocarcinoma) and metastases from bowel or neuroendocrine cancers, which may allow for opportunities for wider clinic benefits at the risk of exacerbating organ shortages and deaths on the waiting list. A more extensive adoption of live donor transplant may be the only option to meet demands. Strategies to reduce the need for solid organ transplant may be essential and may be required to reduce the escalating transplant costs. Cell therapy and tissue engineering approaches are at an early stage in the pathway to clinical translation but will be essential in the quest to manage the increasing burden of liver diseases on society.

## Figures and Tables

**Table 1:** Developing strategies in liver transplant.

Developing Strategies	(Bio)Artificial Liver Device	Organ Reconditioning Centres	Decellularised Scaffolds for Cell Transplant	Synthetic Scaffolds	Pluripotent Stem Cell-Derived Hepatocytes	Gene Therapy	Immunotherapy/Treg Expansion
Stage of development	Clinical	Clinical	Pre-clinical	Pre-clinical	Pre-clinical	Clinical	Clinical
Potential benefits	Prevent patients dying in ALF prior to transplant. Support liver function until native recovery.	Addresses scarcity of organs. Cost-saving. Potential for pharmacological and biological interventions.	Addresses scarcity of donor organs. Rapid and organised development of new livers.	Addresses organ scarcity. Easy to synthesise. Not pathogenic.	Address scarcity of donor organs. Unlimited self-renewal capacity.	Prevent the need for transplantation and lifelong immunosuppression and associated morbidity. Addresses scarcity of organs.	Prevent lifelong immunosuppression and associated morbidity.
Main limitations	Failed to demonstrate survival benefits.	Dependant on the clinical translation of complimentary bioengineering developments.	Requires donor organ. Unable to completely decellularise without damaging ECM, hindering engraftment of cells, lack of micro-re-vascularisation, thrombogenicity and unfavourable host immune response.	Difficult to produce liver microstructure. Poor biocompatibility.	Reprogramming efficiency low. Concern regarding tumorgenicity. Viability	Tumorgenicity concerns. Transient gene expression. High costs. Immune response to viral vectors.	Tumorgenicity concern. Despite success in autoimmune and animal models, it has not translated with success in transplanted organs.

Abbreviations: ECM—Extracellular matrix; ALF—Acute liver failure.
